# Matrine protects colon mucosal epithelial cells against inflammation and apoptosis via the Janus kinase 2 /signal transducer and activator of transcription 3 pathway

**DOI:** 10.1080/21655979.2022.2031676

**Published:** 2022-02-27

**Authors:** Aimei Chen, Defang Fang, Yan Ren, Zhiyong Wang

**Affiliations:** a Lianyungang TCM Branch of Jiangsu Union Technical Institute, Lianyungang, China; b Nancheng Community Health Service Center, Lianyungang, China; c Xinhua Hospital Chongming Branch Gastroenterology, Shanghai, China

**Keywords:** Ulcerative colitis, matrine, apoptosis, JAK2, STAT3 pathway

## Abstract

Ulcerative colitis (UC) is a type of chronic disease of inflammation, and matrine has anti-inflammatory activity. However, it is unclear that whether matrine can alleviate UC. This study aimed to evaluate the effect of matrine on DSS-induced intestinal epithelial cell injury. Cell viability was performed by MTT assay. Then cell apoptosis was analyzed using the TUNEL assay and flow cytometry. The levels of interleukin (IL)-2, IL-6, TNF-α, and IL-1β were evaluated using qRT-PCR. Myeloperoxidase (MPO) activity was detected using ELISA assay. Nitric oxide (NO) production was detected by the Griess reagent. Bax, cleaved caspase-3, Bcl-2, JAK2, p-JAK2, STAT3, p-STAT3, STAT5, p-STAT5 levels were measured by Western blot. Bax (6A7) was asses using immunoprecipitation and immunofluorescence assays. The results illustrated that cell viability was inhibited as the concentration of DSS increased. Matrine did not affect cell viability at the concentration of 0–2 mg/ml but inhibited cell viability in a time-independent manner. Matrine suppressed the levels of pro-inflammatory factors, MPO activity, NO production, and apoptosis of DSS-stimulated cells. Furthermore, we found that matrine inhibited the levels of p-JAK2/JAK2 and p-STAT3/STAT3 but did not affect p-STAT5/STAT5. AG490 treatment further enhanced the effect of matrine on the apoptosis and pro-inflammatory factor levels in DSS-induced cells. In summary, matrine protected NCM460 cell against injury by inactivating the JAK2/STAT3 pathway. These data suggested for the first time that matrine may effective in treating UC.

## Introduction

Ulcerative colitis (UC) is a type of chronic, nonspecific inflammatory disease of the colon. To date, the etiology of UC remains unclear and may be related to environmental, genetic, microbiome, and immune factors [1,2]. The main clinical features of UC are coelialgia, diarrhea, and hemafecia [3]. Longstanding UC may contribute to colorectal cancer, even leading to death [4]. In recent years, the occurrence rate is increasing globally, particularly in developing countries [5]. Aminosalicylates (5-ASA) are the preferred initial treatment for mild-to-moderate UC. Oral glucocorticoid therapy may be considered in patients with an inadequate 5-ASA response. Patients with acute severe UC may require colectomy [6]. Although a variety of drugs are used for UC therapy, this disease is prone to relapse and is associated with long-term side effects [7]. Since the detailed mechanisms involved in the pathogenesis of UC are still unclear, it is essential to find more effective treatment strategies.

Traditional Chinese herb *Sophora flavescens* Ait is contributed to treat UC, especially UC with damp-heat accumulation syndrome from the perspective of traditional Chinese medicine theory [8]. Matrine is a kind of natural alkaloid that isolated from *Sophora flavescens*. It has multiple pharmacological actions, including diuresis, anti-inflammatory activity, anti-tumor, antibacterial effect, and detoxification [9,10]. With further study on the pharmacological effects, matrine has been found to have therapeutic effects in a variety of diseases, such as malignancy [11], inflammatory diseases [12], fibrotic diseases [13], and myocardial injury [14]. A previous has reported that matrine improves the pathological changes of colon tissues and alleviates inflammation [15]. Wu et al. have reported that matrine can improve inflammatory status and oxidative balance in LPS-induced Caco-2 cells [16]. Suo et al. have showed that matrine improves NO-dependent vasomotor and decreases LPS-induced inflammatory cytokines of rat intestinal microvascular endothelial cells [17]. However, how matrine reduces the inflammatory response to UC remains unknown. Thus, it is necessary to further study the function and potential mechanism of matrine in regulating UC phenotype.

The aim of the study was to investigate whether matrine could affect UC and meantime evaluate the potential mechanism. We hypothesized that matrine treatment alleviates inflammation and apoptosis of DSS-induced NCM460 cells. This role was played through JAK2/STAT3 signaling pathway. The goal of this study may provide a new strategy for treating UC

## Materials and methods

### Cell culture

NCM460 cells (human colon mucosal epithelial cell line) were purchased from the cell bank of the Chinese Academy of Sciences (Shanghai, China). All the cells were cultured in DMEM supplemented with 10% FBS, 100 U/mL penicillin, and 100 μg/mL streptomycin (All obtained from Gibco, Grand Island, USA) under the condition of 37°C with 5% CO_2_.

Dextran sulfate sodium (DSS; purity >98%) was purchased from Amresco (Solon, USA). NCM460 cells in the logarithmic growth stage were plated in six-well plates until cell confluence attained 80%. The cells were treated with 0%, 0.5%, 1%, 2%, and 4% DSS for 24 h.

### Matrine treatment

**Figure 1.**
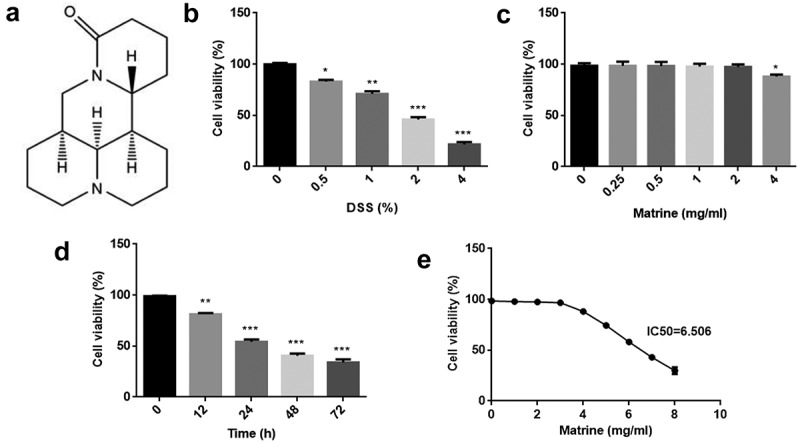
Effects of matrine on cell viability of NCM460 cells. (a) Chemical structure of matrine. (b) Cell viability was measured using MTT assay after NCM460 cells treating with 0, 0.5, 1, 2, and 4% DSS for 24 h. (c) Cell viability was assessed using MTT assay after treating with 0, 0.25, 0.5, 1, 2, and 4 mg/ml matrine for 24 h. (d) Cell viability was assessed using MTT assay after the cells treating with 2 mg/ml matrine for 0, 12, 24, 48 and 72 h. (e) Matrine IC50 was calculated. *P < 0.05. **P < 0.01. ***P < 0.001.

Matrine (purity >98%; Figure 1(a)) was purchased from Sbjbio (Nanjing, China). After treating with DSS or sterile water, NCM460 cells were exposed to series concentration of matrine (0, 0.25, 0.5, 1, 2, and 4 mg/ml) for 24 h. Then, an optimal concentration of matrine was utilized to treat the cells for 0, 12, 24, 48, and 72 h. To detect matrine IC50, NCM460 cells were exposed to 0, 1, 2, 3, 4, 5, 6, 7, and 8 mg/ml for 24 h.

### AG490 treatment

JAK2 inhibitor (AG490; purity >99%) was purchased from Abcam (Cambridge, USA). AG490 (0, 1, 2.5, 5, 10, 20, and 40 μM) was used to treat cells for 24 h.

### MTT assay

Cell viability was evaluated using MTT assay. In brief, the cells were incubated with MTT (10 μl; Procell, Wuhan, China) at 37°C for 4 h after matrine treatment. Then, DMSO (150 μl) was incubated with the cells for 10 min to dissolve the formazan. The absorption was tested by a microplate reader (Bio-Rad, Hercules, USA) at 490 nm.

### TUNEL assay

TUNEL assay was carried out using TUNEL Apoptosis Detection kit (Yeasen, Shanghai, China). After washing, the cells were fixed with paraformaldehyde for 20 min on ice. Permeabilization was performed using 0.2% Triton X-100 at 25°C for 5 min. Then, the cells were incubated with TdT reaction buffer containing Alexa Fluor 488–12-dUTP Labeling Mix at 37°C for 60 min. At last, stained cells were visualized and photographed using a fluorescence microscopy (Olympus, Tokyo, Japan).

### Flow cytometry

After matrine treatment, 2х10^5^ cells were collected and resuspended using 1х binding buffer (100 μl) in the centrifuge tubes. Annexin V-PE (5 μl) and 7-AAD staining solution (10 μl) in an Annexin V-PE/7-AAD Apoptosis Detection Kit (Yeasen) were incubated with the cells for 15 min. After adding 1х binding buffer (400 μl), flow cytometry was performed within 1 h using a CytoFLEX flow cytometer (Beckman Coulter, Fullerton, USA).

### qRT-PCR

Trizol reagent (Invitrogen, Carlsbad, USA) was utilized for total RNA collection. PrimeScript RT Master Mix Kit and TB Green Premix Ex Taq II Kit (Takara, Beijing, China) are used for RT-PCR. GAPDH acted as an endogenous control. The relative expression was analyzed using the 2^−ΔΔCT^ method. The sequences of specific primers are shown in Table 1.

**Table 1.** Sequences of the specific primers used in qRT-PCR

Name	Forward (5’-3’)	Reverse (5’-3’)
TNF-α	CCC*ACTCTGACCCCTTTACT*	TTT*GAGTCCTTGATGGTGGT*
IL-1β	CATCTTTGAAGAAGAGCCCG	AACTATGTCCCGACCATTGC
IL-2	AAGGAAACACAGCAGCACCT	CACAGTTGCTGGCTCATCAT
Il-6	CCGGAGAGGAGACTTCACAG	GAGCATTGGAGGTTGGGGTA
GAPDH	CTG*GAGAAACCTGCCAA*GTA	TGT*TGCTGTAGCCGTATTCA*

### ELISA assay

Cells were centrifuged at 10,000 × g for 5 min. Myeloperoxidase (MPO) activity was detected in the supernatant using MPO ELISA kit (Cusabio, Wuhan, China) according to the manufacturer’s protocol [18].

### Nitric oxide (NO) production

The level of NO production was detected using Griess reagent kit (Abcam) [19]. Cells were centrifuged at 10,000 × g for 5 min, and the supernatant was transferred to the new tube. Then, supernatant (100 μl) was mixed with Griess reagent (100 μl). OD value at 540 nm was measured using microplate reader.

### Western blot

The cells were lysed using RIPA buffer (Sigma-Aldrich, St. Louis, USA). After quantifying with a BCA kit (Sigma-Aldrich), 30 µg denatured protein was added per lane to separate using 10% SDS-PAGE. The protein was transferred to PVDF membranes, blocked with 5% nonfat milk for 1 h, and incubated with primary antibodies overnight at 4°C. The secondary antibody (ab205718, 1:5000; Abcam) was incubated with the cells the next day for 1 h. The bands on the membranes were visualized using ECL reagent (Sigma-Aldrich). Image J software was utilized for gray analysis.

### Immunoprecipitation (IP) assay

To detect active Bax (Bax (6A7)), the cells were lysated, and then incubated with immunoprecipitated with protein A agarose beads (Cell Signaling Technology, Danvers, MA, USA) and the conformation-specific primary antibody 6A7 (Santa Cruz Biotechnology, Santa Cruz, CA, USA). At last, the protein samples were measured by Western blot.

### Immunofluorescence (IF) assay

The active form of Bax was also analyzed using IF assay [20]. BCM460 Cells were fixed with 4% paraformaldehyde. Then, the cells were incubated with 5% normal goat serum and 0.1% Triton X-100. After incubating with anti-6A7 Bax (BD Pharmingen) at 4°C overnight, the cells were incubated with Alexa fluor 488 goat anti-mouse IgG at room temperature for 1 h. The images were visualized using laser scanning confocal microscope.

### Statistical analysis

Three replicates were set for every experiment. Mean ± SD was analyzed by GraphPad Prism 7 (La Jolla, USA). Unpaired Student's t-test was conducted to evaluate differences between two groups. One-way ANOVA was used to compare differences among multiple groups. P < 0.05 declared statistically significant difference.

## Results

The study aimed to explore the role of matrine in UC. We used DSS to establish the cell injury model. We assessed cell apoptosis and the expression of inflammatory factors. We hypothesized that matrine inhibited inflammation and apoptosis of DSS-induced NCM460 cells by the JAK2/STAT3 pathway. The study provided a reference for the clinical application of matrine in UC.

### Toxicity of matrine to NCM460 cells

First, we used different concentrations of DSS to establish cell injury model. Cell viability was reduced in a dose-dependent pattern (Figure 1(b)). NCM460 cells were treated 0, 0.25, 0.5, 1, 2, and 4 mg/ml matrine for 24 h, and we found matrine did not affect cell viability at 0–2 mg/ml concentration, but inhibited cell viability at 4 mg/ml concentration (Figure 1(c)). Then, 2 mg/ml matrine was used to treat cells for 0, 12, 24, 48, and 72 h. The data showed that matrine reduced cell viability in a time-dependent manner. There was a significant difference between 0 and 24, 48, or 72 h, rather than 0 and 12 h (Figure 1(d)). Moreover, matrine IC50 value was 6.506 (Figure 1(e)).

### Matrine inhibited inflammation in DSS-induced cells

**Figure 2.**
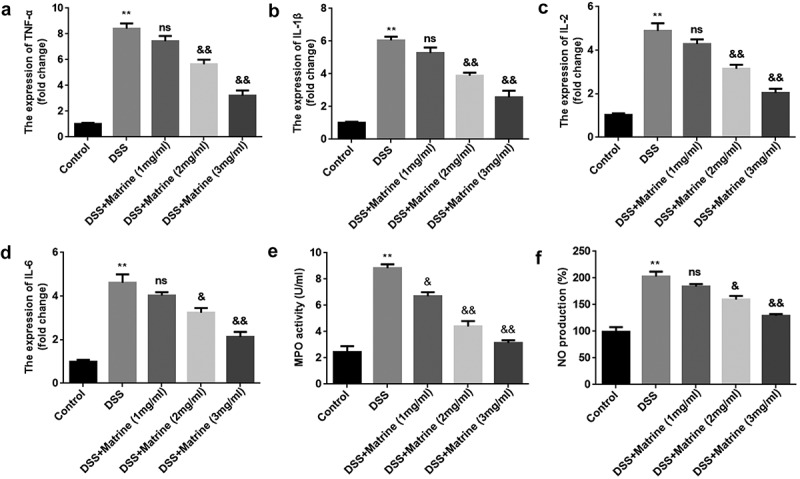
Matrine reduced the release of inflammatory factors in NCM460 cells stimulated by DSS. After DSS-stimulated NCM460 cells treating with 1, 2, and 3 mg/ml matrine, the expression of (a) TNF-α, (b) IL-1β, (c) IL-2, and (d) IL-6 was detected using qRT-PCR, (e) MPO activity was detected using ELISA assay, and (f) NO production was detected using Griess reagent. **P < 0.01 vs. the control group. &P < 0.05 and &&P < 0.01 vs. the DSS group. ns: no significant difference.

To further study the role of matrine in UC, DSS-stimulated cells were treated with 1, 2, and 3 mg/ml matrine. The expression of pro-inflammatory factors (TNF-α, IL-1β, IL-2, IL-6) was significantly elevated by DSS, while matrine significantly reversed the elevation with the concentration increase (Figure 2(a-d)). Additionally, matrine suppressed DSS-induced MPO activity and NO production (Figure 2(e,f)). Thus, the following study used 2 mg/ml matrine to treat NCM460 cells.

### Matrine reduced DSS-induced cell apoptosis

**Figure 3.**
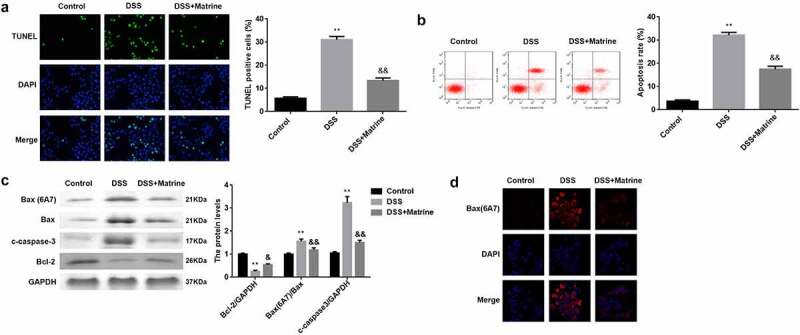
Matrine suppressed DSS-induced apoptosis of NCM460 cells. Cell apoptosis was assessed using (a) the TUNEL assay, and (b) flow cytometry. (c) The Bcl-2, Bax (6A7), Bax, and cleaved caspase-3 expression was measured by Western blot. (d) Bax (6A7) was examined using IF assay. **P < 0.01 vs. the control group. &P < 0.05 and &&P < 0.01 vs. the DSS group.

Then, we explored that how does matrine reduces inflammation response. The results of the TUNEL assay and flow cytometry illustrated that DSS significantly facilitated cell apoptosis. Matrine treatment significantly abolished the facilitation of apoptosis of DSS-induced cells (Figure 3(a,b)). Moreover, the Bax (6A7), Bax, and cleaved caspase-3 levels were markedly elevated, but Bcl-2 levels were markedly decreased in the DSS group. However, matrine treatment obviously rescued the effects induced by DSS (Figure 3(c)). In addition, DSS markedly enhances its ability to bind antibodies to 6A7, while matrine markedly rescued the enhancement (Figure 3(d)).

### Matrine inhibited the activation of the JAK2/STAT3 pathway

**Figure 4.**
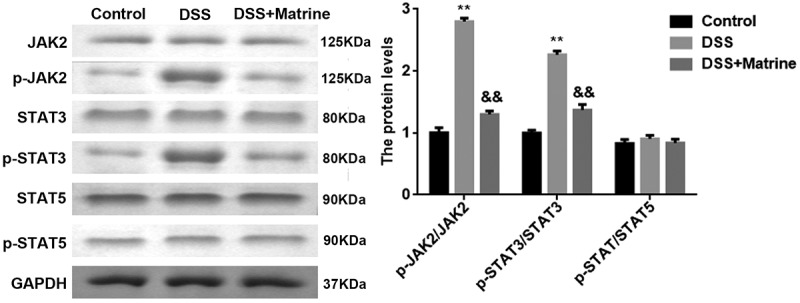
Effect of matrine on JAK2/STAT3 pathway. The JAK2, p-JAK2, STAT3, p-STAT3, STAT5, and p-STAT5 expression was tested using Western blot. **P < 0.01 vs. the control group. &&P < 0.01 vs. the DSS group.

As shown in Figure 4, the protein levels p-JAK2 and p-STAT3 were significantly upregulated by DSS, which were rescued by matrine. However, the JAK2, STAT3, STAT5, and p-STAT5 levels were not affected by DSS and matrine (Figure 4).

### Matrine suppresses DSS-induced cell apoptosis via the JAK2/STAT3 pathway

**Figure 5.**
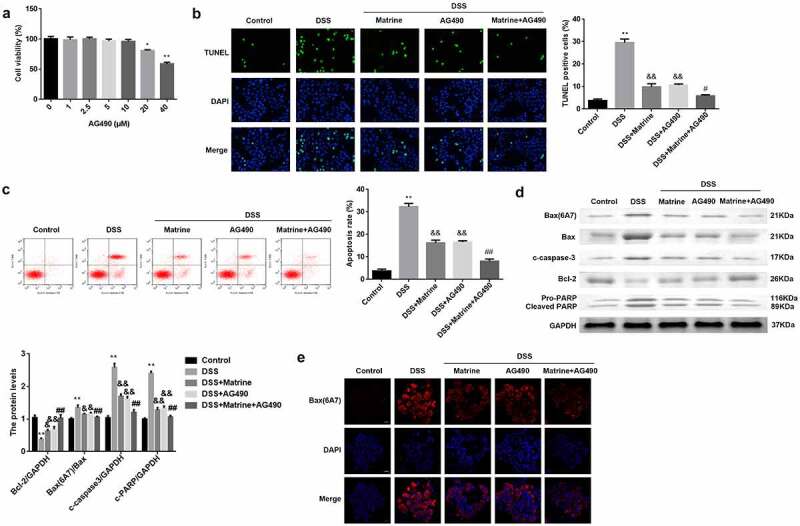
Matrine suppresses DSS-induced NCM460 cell apoptosis via the JAK2/STAT3 pathway. (a) Cell viability was measured using MTT assay after NCM460 cells treating with 0, 1, 2.5, 5, 10, 20, and 40 μM for 24 h. Cell apoptosis was assessed using (b) the TUNEL assay, and (c) flow cytometry. (d) Western blot was conducted to analyze the expression of apoptotic related factors including Bax (6A7), Bcl-2, Bax, cleaved caspase-3, and PARP. (e) Bax (6A7) was examined using IF assay. **P < 0.01 vs. the control group. &P < 0.05 and &&P < 0.01 vs. the DSS group. #P < 0.05 and ##P < 0.01 vs. the DSS + Matrine group.

To verify the potential mechanism, AG490 was utilized to treat the cells to inhibit the JAK2/STAT3 pathway. AG490 (0–10 μM) did not affect cell viability, and 20 and 40 μM AG490 significantly inhibited cell viability (Figure 5(a)). The promotion of cell apoptosis induced by DSS was significantly reversed by matrine or AG490. Furthermore, AG490 inhibited apoptosis in DSS-stimulated cells treated with matrine (Figure 5(b,c)). Matrine or AG490 markedly downregulated Bax (6A7), Bax, cleaved caspase-3, and cleaved PARP in DSS-stimulated cells, while AG490 enhanced the effects induced by matrine (Figure 5(d)). Inversely, Bcl-2 levels were obviously decreased by DSS, and significantly rescued by matrine or AG490. Moreover, AG490 obviously elevated the Bcl-2 levels, compared with DSS + matrine group (Figure 5(d)). As shown in Figure 5(e), matrine significantly inhibits its ability to bind to 6A7 antibodies, while AG490 markedly enhanced matrine’s effect.

### Matrine alleviates DSS-induced inflammation through the JAK2/STAT3 pathway

**Figure 6.**
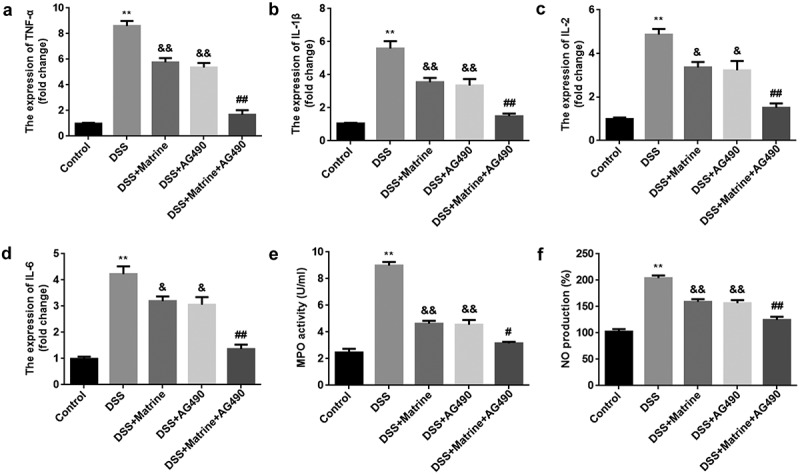
Matrine alleviates DSS-induced inflammation in NCM460 cells through the JAK2/STAT3 pathway. After DSS-induced NCM460 cells treating with matrine and AG490, the expression of (a) TNF-α, (b) IL-1β, (c) IL-2, and (d) IL-6 was detected using qRT-PCR, (e) MPO activity was detected using ELISA assay, and (f) NO production was detected using Griess reagent. **P < 0.01 vs. the control group. &P < 0.05 and &&P < 0.01 vs. the DSS group. #P < 0.05 and ##P < 0.01 vs. the DSS + Matrine group.

At last, the pro-inflammatory factor levels were tested. AG490 or matrine significantly reduced the mRNA levels of TNF-α, IL-1β, IL-2, and IL-6 in DSS-stimulated cells, and AG490 further enhanced the effects induced matrine (Figure 6(a-d)). Moreover, matrine markedly inhibited MPO activity and decreased NO production, while AG490 promoted the effect of matrine (Figure 6(e,f)).

## Discussion

In the present study, we studied whether matrine could affect UC. Matrine treatment can reduce the release of pro-inflammatory factors and inhibit cell apoptosis. Moreover, the role of matrine is played via modulating the JAK2/STAT3 pathway.

UC is difficult to cure completely. At present, the drugs commonly used to treat UC include hormones, immunosuppressants, and aminosalicylic acid preparation. However, high side effects of these drugs are existing and are prone to drug resistance [21]. With the development of the research on Chinese herbal medicine, we found some active ingredients that can ameliorate UC. For example, chlorogenic acid reduces the damage to the colonic mucosa and suppresses inflammatory response [22]. Rhein decreases uric acid by altering purine metabolism, thus alleviating DSS-induced UC [23]. Baicalin regulates Treg/Th17 balance, oxidative stress, and gut microbiota to protect rats against UC [24]. Arbutin ameliorated DSS-induced UC of mice [18]. Matrine, as an anti-inflammatory alkaloid, alleviates intestinal inflammation induced by LPS [16]. However, whether matrine can affect UC is unknown. IL-2, IL-6, TNF-α, and IL-1β factors have a pro-inflammatory effect, in which levels are elevated in the colon tissue of UC [25]. Studying the expression of these pro-inflammatory factors can help to assess UC activity [26]. In this study, we found that matrine reduced the release of these pro-inflammatory factors, MPO activity, and NO production in DSS-stimulated cells. The results suggest that matrine can protect NCM460 cell against DSS-induced inflammation, consistent with the results in vivo [27].

Cell apoptosis plays an essential role in gut tissue homeostasis and the regeneration of intestinal epithelial cells. Excessive apoptosis is closely correlated to the inflammatory status of UC [28]. Cleaved caspase-3 is important in the process of apoptosis. Bcl-2 and Bax, membranes of Bcl-2 family, have anti- and pro-apoptotic effects, respectively [29]. It has been reported that the activation of cytokines such as TNF and IL results in apoptosis of intestinal epithelial cells [30]. Several previous studies have revealed that inhibition of apoptosis contributes to alleviating UC [31,32]. Additionally, matrine treatment regulates apoptosis of several kinds of cells, such as cardiomyocytes, myoblasts, and tumor cells [33–35]. In the present study, DSS promoted cell apoptosis, while matrine suppressed apoptosis of DSS-treated cells. Meanwhile, the levels of apoptotic-related factors were regulated by matrine in DSS-induced cells. The findings suggest that matrine suppressed apoptosis of intestinal epithelial cells.

The advantage of matrine to treat UC mainly includes low drug resistance and small side effects. However, since there are few studies on matrine, and most studies only focus on the cellular level, more in vivo studies are needed before it can be used in clinical trials.

JAK2/STAT3 signaling pathway is associated with several cellular processes, including cell growth, death, and inflammatory response [36]. JAK2, a member of the JAK family, is regulated by cytokines. Activating JAK2 promotes the phosphorylation of STAT3, subsequently resulting in STAT3 activating [37]. Abnormal activation of the JAK2/STAT3 pathway is closely involved in numerous inflammatory diseases progression, including arthritis, hepatitis, nephritis, and UC [38–40]. A previous study has shown that sphk1 makes a deterioration of UC by activating JAK2/STAT3 pathway [41]. Butyric acid alleviates UC via inactivating JAK2/STAT3 pathway [42]. Furthermore, JAK2 is related to the proliferation and apoptosis of young adult mouse colon cells [43]. In this study, we explored the underlying mechanism of matrine in UC. The ratio of p-JAK2/JAKs and p-STAT3/STAT3 was reduced by matrine. Moreover, AG490, the JAK2 inhibitor, inhibited apoptosis, and downregulated pro-inflammatory factor levels in DSS-induced cells. Furthermore, the effect of matrine in DSS-induced cells was further enhanced by AG490 treatment, suggesting that matrine ameliorates UC by inactivating JAK2/STAT3 signaling pathway. The effect of AG490 is more significant than that of matrine, suggesting that the regulation of the JAK2/STAT3 pathway is more direct than matrine to DSS-induced NCM460 cells.

The main innovation of this study is that we found for the first time that matrine protects intestinal epithelial cells from injury by inactivating JAK2/STAT3 pathway. However, the study still has limitations. We have not done *in vivo* studies on the effects of matrine. Thus, the clinical significance of matrine remains largely unknown. Our future work may focus on the role of matrine *in vivo*.

## Conclusion

Matrine protected the intestinal epithelial cells from injury by inhibiting cell apoptosis and suppressing the release of pro-inflammatory factors through inactivating JAK2/STAT3 pathway. This study is the first to provide a theoretical basis for matrine to become a therapeutic drug for UC.

## Research Highlights

1. Matrine reduced pro-inflammatory factors and apoptosis of DSS-induced NCM460 cells.
2. Matrine inhibited the activation of the JAK2/STAT3 pathway induced by DSS.
3. Matrine protected NCM460 cells against injury by regulating the JAK2/STAT3 pathway.
